# Prevalence and correlates of anemia among adolescents in Nepal: Findings from a nationally representative cross-sectional survey

**DOI:** 10.1371/journal.pone.0208878

**Published:** 2018-12-14

**Authors:** Binaya Chalise, Krishna Kumar Aryal, Ranju Kumari Mehta, Meghnath Dhimal, Femila Sapkota, Suresh Mehata, Khem Bahadur Karki, Donya Madjdian, George Patton, Susan Sawyer

**Affiliations:** 1 Nepal Health Research Council, Kathmandu, Nepal; 2 Helen Keller International, Kathmandu, Nepal; 3 Ministry of Health and Population, Kathmandu, Nepal; 4 Department of Social Sciences, Sociology of Consumption and Households, Wageningen University, Wageningen, the Netherlands; 5 Department of Paediatrics, the University of Melbourne, Melbourne, Australia; 6 Centre for Adolescent Health, Royal Children’s Hospital and Murdoch Children’s Research Institute, Melbourne, Australia; University of Bergen, NORWAY

## Abstract

Anemia is regarded as major public health problem among adolescents in Low and Middle-Income Countries (LMICs) but there is limited primary data in many countries, including Nepal. This study investigated the prevalence and correlates of anemia in a nationally representative sample of adolescents within the 2014 National Adolescent Nutrition Survey in Nepal. A total of 3780 adolescents aged 10 to 19 years were selected from a cross-sectional survey through multi-stage cluster sampling. Structured interviews, anthropometric measurements and hemoglobin assessments of capillary blood were obtained. Bivariate and multivariable analyses were undertaken to compute the Adjusted Odds Ratio (aOR) for socio-demographic, behavioral and cluster characteristics. The overall prevalence of anemia was 31% (95%CI: 28.2, 33.5), 38% (95%CI: 34.0, 41.8) in female and 24% (95%CI: 20.6, 27.1) in male. The likelihood of anemia was significantly higher among older adolescents (aOR 1.75, 95%CI: 1.44, 2.13), females (aOR 2.02; 95%CI: 1.57, 2.60), among those who walk barefoot (aOR 1.78, 95%CI: 1.08, 2.94), and those residing in the Terai (aOR 1.80, 95%CI: 1.18, 2.77). Food consumption from more than four food groups (aOR 0.71, 95%CI: 0.57, 0.88) was protective against anemia. In conclusion, anemia is common in Nepali adolescents. Efforts to improve the nutritional status of this high-risk age group require nutrition that focus on eating habits, sanitation, iron supplementation and the treatment of hookworm infection.

## Introduction

Micronutrient deficiency resulting in disorders such as anemia commonly affects adolescents in developing countries. Around one-quarter of adolescents in developing countries are anemic [1], but prevalence estimates for adolescent anemia in the South-East Asia region range from 27% to 55% [2]. Adolescents’ vulnerability to anemia is commonly attributed to the biological demands for micronutrients (such as iron and folic acid) associated with rapid physical growth, as well as from loss of these micronutrients due to parasitic infestations like malaria and hookworm [3]. At the end of adolescence, male rapidly regain adequate nutrient stores, whereas female remain vulnerable to anemia as a result of menstrual blood loss. They may therefore continue to be anemic or become more anemic because of increased micronutrient requirements from menstruation as well as from pregnancy and lactation [4].

Anemia not only adversely affects adolescents’ physical growth, but it also hinders them achieving their full potential by diminishing educational achievement and labor productivity [2]. Previous research has shown adverse effects of anemia on adolescents’ cognitive function and mental health, as well as lower school attendance, learning, academic achievements and decreased work performance [5–8]. In pregnant girls, anemia increases the risk for birth complications and delivery of low birth-weight infants. Hence, anemia is not only a concern for today’s adolescents, but also for society’s future development [9].

In the context of LMICs including Nepal, there is scarce evidence on micronutrient deficiencies among adolescents and in particular, around anemia in adolescents [10]. The Nepal Demographic and Health Survey (NDHS) is a primary source of national data on anemia, but it explicitly provides information on only mothers and children under-five years old. According to the NDHS 2011, 46% of children aged 6–59 months, and 35% of women aged 15–49 years were anemic [11]. Although there has been a substantial reduction of anemia among women in Nepal between 2001 and 2006, but improvements have stagnated since 2006. Several small-scale studies have attempted to examine the prevalence of anemia in adolescents with alarming results. For example, a survey of 308 adolescents aged 10 to 19 years from the Morang district in Nepal showed an overall prevalence of adolescent anemia of 66% [12]. Hospital-based studies conducted among 10 to 19 year old adolescents showed an overall prevalence of 52% - 56% [13, 14]. However, these small studies were each limited to a local region. We designed the current study to determine the prevalence of adolescent anemia and its correlates within a larger nationally representative sample (n = 3780) of 10 to 19 year olds in Nepal.

## Methods

### Study site and sampling

At the time of the survey, Nepal was administratively divided into five development regions, 14 Zones and 75 Districts. Each district was further divided into Village Development Committees (VDCs) and municipalities [15]. Geographically, Nepal consists of three regions reflecting mountains (Himal), hills (Pahad) and the lowland (Terai) regions. A nationally representative cross-sectional survey was conducted in 2014 among male and female (non-pregnant) adolescents aged 10 to 19 years old. We used a stratified cluster sampling method [16] to select 3780 adolescents from 13 districts of Nepal.

A three-staged cluster sampling was performed within each stratum. The first stage of cluster sampling involved a random selection of 13 districts. For this purpose, we divided the entire country into 13 sub-regions as described in the 2011 NHDS [11]. One district was randomly selected from each of the 13 sub-regions. This resulted in three districts being selected from the mountainous and five each from the hilly and lowland regions. The total number of strata was six, two (male and female) in each from three ecological regions. The second stage involved the selection of clusters from these districts. A total of 90 VDCs and municipalities (clusters) were chosen using the Probability Proportion to Size (PPS) sampling from the total VDCs and municipalities within those 13 selected districts. Within each chosen cluster, we selected a fixed number of 21 male and 21 female adolescents through a systematic sampling of households. We randomly selected an eligible participant if there was more than one eligible participant in the selected household.

The sample size was determined considering the national prevalence of anemia of 39% and an allowable error of 5%. A detail description of sample size calculation is reported elsewhere [17]. The initial sample size was 365. Considering a design effect of 1.5 and multiplying the size by 6 (total number of strata), the sample size was 3290. We obtained the final sample size of 3655 after adjusting for a non-response rate of 10%. We then rounded up to 3780 to ensure an equal number of male and female participants from each stratum. Altogether, 3762 adolescents participated in the study. A detailed description of the sampling method is reported elsewhere [17].

### Data collection

Trained enumerators conducted one-on-one interviews with adolescents using a pretested structured questionnaire, which covered information on socio-demographic characteristics, sanitation and nutrition. Participant height and weight was measured using stadiometer and digital weighing scale, respectively. Blood hemoglobin concentration was measured using the HemoCue method. Trained laboratory professionals conducted a procedure to collect capillary blood samples after obtaining written consent from both the adolescents and their parents. A sterile lancet was used to prick blood from a finger. After discarding the first two drops, the third and fourth drops of blood were collected on a microcuvette. The microcuvette was then placed in a HemoCue machine after calibrating it to zero. Laboratory professionals referred HemoCue operation manuals for storage of microcuvette and analysis of blood samples.

### Study variables

The primary outcome was anemia. We adjusted the measured hemoglobin concentrations by the residential elevation (altitude) of participants. Nepal’s altitude varies between 70 to 8848 meters above sea level. The maximum residential altitude of participants was up to 2740 meters, with 50% of participants residing at higher than 1000 meters above sea level. We obtained the altitude adjusted haemoglobin concentration among participants living at altitude higher than 1000 meters by deducting the WHO recommended adjustment values [18] to the measured haemoglobin concentration. Subsequently, we used age and sex-specific cut-offs as defined by the WHO [18] to categorize the adjusted hemoglobin values as anemic and non-anemic adolescents. The detail description of adjustment method is reported elsewhere [17]. The independent variables were socio-demographic factors (age, sex, marital status, religion, ethnicity, family structure, family size), economic factors (household income, main source of family income), contextual factors (place of residence, ecological region), sanitation factors (households having latrine, adolescents walking barefoot) and nutrition factors (BMI, dietary diversity). Categorization of ethnicity was based on the caste system in Nepal, which consists of more than 125 groups. Based on the socioeconomic status and cultural hierarchy of these groups in Nepalese society [19, 20], they were first grouped into six groups namely: upper caste groups, relatively advantaged janajati, religious minorities, disadvantaged non-dalit Terai cast, disadvantaged janajati and dalits. For the purpose of this study, we further divided these into two broad groups: advantaged ethnic groups (consisting of upper caste groups and relatively advantaged janajati) and disadvantaged groups (all others). Religion was broadly classified as Hindus and non-Hindus. Family structure was categorized into nuclear or extended family system based on the living arrangement/household composition of adolescents. A nuclear family consisted of a couple living with their children, whereas an extended family included other relatives related by blood or marriage [21]. Participants were referred to as walking barefoot if they did not wear shoes while outdoors. We computed Body Mass Index (BMI) Z-scores of the adolescents and used the WHO Growth Reference cut-offs [22] to categorize the Z-scores into thinness (less than -2 SD), normal (-2 SD to +1 SD) and overweight (greater than +1SD). Dietary diversity was determined by asking the adolescents if they had consumed a number of food groups (such as dairy products, grains, fruits and vegetables, eggs, meat and poultry, fish, legumes and nuts) within the 24 hours preceding the survey. We counted and summed the number of food groups consumed over 24 hours [23].

### Statistical analysis

Data was processed into EpiData (version 3.1) and then exported into STATA version 15 for further analysis. We conducted complex survey analyses, using STATA survey command to estimate anemia prevalence at a 95% Confidence Interval (CI). We used Chi-square tests for bivariate analysis to assess associations between the independent variables and adolescent anemia. *P-values* of less than 0.05 were considered as statistically significant. Covariates were considered for the logistic regression if the *p-value* was less than 0.25 in the bivariate analysis. We assessed multicollinearity of the covariates using Variance Inflation Factors (VIFs). Covariates with VIFs of more than 0.2 were also excluded from the logistic regression analysis [24]. We used multivariable logistic regression adjusting all eligible correlates (age, sex, marital status, religion, ethnicity, household latrine, walking barefoot, BMI, ecological zone, place of residence and umber of food groups consumed) included in the model.

### Ethical considerations

The Ethical Review Board of the Nepal Health Research Council reviewed the research proposal and provided ethical approval for this study. Informed written consent was obtained from research participants and their parents/guardians prior to data collection.

## Results

### Anemia prevalence

Table 1 shows the status of anemia among adolescents by socio-demographic, behavioral and cluster characteristics. Overall, 31% (95%CI: 28.2, 33.5) of adolescents were anemic at the time of the study. The prevalence was particularly high among 15 to 19 year olds (37%, 95%CI: 33.5, 40.6) and female adolescents (38%, 95%CI: 34.0, 41.8) compared to young (10–14 years) and male adolescents, respectively. Anemia prevalence was also high among Hindus (31%, 95%CI: 28.4, 34.0) and the disadvantaged ethnic groups (34%, 95%CI: 30.7, 37.0) compared to non-Hindus and the elite ethnic groups. Adolescents living in the lowlands (Terai) had the highest prevalence of anemia (38%, 95%CI: 33.8, 41.4) compared to hills (24%, 95%CI: 20.4, 28.4) and the mountains (23%, 95%CI: 16.9, 30.0) regions. Forty-eight percent (95%CI: 36.2, 60.0) of adolescents who walked barefoot were anemic, compared to 30% of adolescents who wore shoes (95%CI: 27.4, 32.5). Anemia was common among those with less diversity in their diets. For example, 34% (95%CI: 30.8, 36.3) of adolescents who consumed food from less than four food groups were anemic compared to 25% of those consuming meals consisting of more than four food groups per day (95%CI: 20.0, 28.8).

**Table 1:** Prevalence of anemia among adolescents in Nepal obtained from National Adolescent Nutrition Survey 2014.

	Adolescent Anemia			
	Total No	Frequency	Prevalence	95% CI
Age Years***				
Younger Adolescents (10–14)	2273	497	26.7	23.6, 29.9
Older Adolescents (15–19)	1489	465	37.0	33.5, 40.6
Sex***				
Male	1881	378	23.7	20.6, 27.1
Female	1881	584	37.8	34.0, 41.8
**Marital Status**				
Unmarried	3658	925	30.5	27.9, 33.2
Married	104	37	40.6	30.0, 52.0
**Religion**				
Non-Hindus	665	144	27.8	23.0, 33.1
Hindus	3097	818	31.1	28.4, 34.0
Ethnicity***				
Advantaged	1691	350	25.8	22.4, 29.5
Disadvantaged	2071	612	33.8	30.7, 37.0
**Family Structure**				
Nuclear	2523	632	31.0	28.0, 34.2
Extended	1239	330	30.2	26.6, 34.1
**Family Size**				
< 5 Members	752	173	29.3	24.8, 34.1
≥ 5 Members	3010	789	31.1	28.3, 34.0
**Household Latrine**				
Yes	3032	743	29.6	26.8, 32.5
No	730	219	33.6	29.0, 38.6
Walks Barefoot***				
No	3644	914	29.9	27.4, 32.5
Yes	118	48	48.0	36.2, 60.0
**BMI**				
Normal	3138	805	30.8	28.0, 33.7
Thin	391	112	34.2	28.4, 40.5
Overweight	182	37	24.9	17.6, 34.1
**Source of Family Income**				
Agriculture	1647	428	31.5	27.8, 35.5
Non-Agriculture	2115	534	30.2	27.4, 33.1
Ecological Zone***				
Mountains (Himal)	1255	214	22.8	16.9, 20.0
Hills (Pahad)	1253	282	24.2	20.4, 28.4
Lowlands (Terai)	1254	466	37.6	33.8, 41.4
**Place of Residence**				
Urban	273	88	35.8	27.2, 45.3
Rural	3489	874	30.1	27.4, 33.0
**Family Income**				
NPR ≤2000	163	34	27.4	17.8, 39.7
NPR 2001–5000	911	225	30.7	26.8, 34.8
NPR 5001–10000	1224	302	30.3	26.6, 34.1
NPR >10000	1463	401	31.5	28.0, 35.1
No of Food Groups Consumed***				
<4	2518	716	33.5	30.8, 36.3
≥4	1244	246	24.7	20.0, 28.8
**Total**	**3762**	**962**	**30.7**	**28.2, 33.5**

* = P < 0.05.

** = P < 0.01.

*** = P < 0.001.

### Correlates of anemia

Table 2 depicts correlates of adolescent anemia obtained by using multivariable logistic regression. Multivariable analysis revealed that the likelihood of anemia was higher among older adolescents (aOR 1.75, 95%CI: 1.44, 2.13) and females (aOR 2.02, 95%CI: 1.57, 2.60) when compared to younger adolescents and males, respectively. Regarding ecological region, a significant association was observed for those living in the Terai (aOR 1.80, 95%CI: 1.18, 2.77) compared with those living in the mountains. Adolescents who walked barefoot (aOR 1.78, 95%CI: 1.08, 2.94) were more likely to be anemic than those who wore shoes while outdoors. In contrast, anemia was less likely among those who consumed four or more food groups (aOR 0.71, 95%CI: 0.57, 0.88) compared to those who ate less than four food groups per day. No clear associations were found between adolescent anemia and marital status, religion, family size, presence of household latrine, weight status, or place of residence (urban versus rural).

**Table 2:** Multivariable analysis of adolescent anemia.

	Crude OR	95%CI		aOR	95%CI
**Age Years (Ref: 10–14)**					
15–19	1.61	1.33, 1.95		1.75	1.44, 2.13
**Sex (Ref: Male)**					
Female	1.95	1.53, 2.49		2.02	1.57, 2.60
**Marital Status (Ref: Unmarried)**					
Married	1.56	0.97, 2.50		1.23	0.75, 2.02
**Religion (Ref: Non-Hindus)**					
Hindus	1.17	0.90, 1.53		1.03	0.76, 1.60
**Ethnicity (Ref: Advantaged**					
Disadvantaged	1.47	1.20, 1.81		1.15	0.93, 1.43
**Household Latrine (Ref: Yes)**					
No	1.21	0.95, 1.52		0.82	0.62, 1.07
**Walked Barefoot (Ref: No)**					
Yes	2.16	1.34, 3.49		1.78	1.08, 2.94
**BMI (Ref: Normal)**					
Thin	1.17	0.87, 1.56		1.20	0.87, 1.66
Overweight	0.75	0.48, 1.16		0.84	0.54, 1.50
**Ecological Zone (Ref: Mountains)**					
Hills (Pahad)	1.08	0.70, 1.67		1.03	0.67, 1.59
Lowlands (Terai)	2.04	1.36, 3.07		1.80	1.18, 2.77
**Place of Residence (Ref: Urban)**					
Rural	0.77	0.51, 1,18		0.83	0.61, 1.14
**No of Food Groups Consumed (Ref: < 4)**					
≥ 4	0.65	0.54, 0.79		0.71	0.57, 0.88

Ref = Reference Group, OR = Odds Ratio, aOR = Adjusted Odds Ratio Ref: Reference Group

## Discussion

This is the first national survey to investigate adolescent anemia in Nepal. It reveals that the overall prevalence of anemia among adolescents aged between 10 and 19 years was 31%. This is lower when compared to prevalence rates (between 52 and 66%) reported in previous studies from Nepal [12–14], which were limited to small-scale hospital based surveys. The risk of anemia was greater in females, older adolescents and adolescents from the lowlands and lower in adolescents consuming food from multiple food groups. The findings provide a national estimate of adolescent anemia, which will serve as a benchmark to evaluate current nutrition programs as well as serve to estimate the National Burden of Disease for Nepal. This finding also provides insights into the determinants of anemia in adolescents within the context of countries like Nepal that have lacked reliable primary data at the national level.

In developing countries, major drivers of malnutrition, including nutrition-related anemia, include family poverty as well as one’s social position within a household. Lack of education, early marriage and childbearing during adolescence may adversely affect girls’ nutritional status. Especially in South Asia, gender inequality and the resulting lack of control by women and girls over their lives, can be an important underlying determinant of malnutrition in general and access to iron-rich foods [25]. Rapid physical growth in adolescence, menarche and resulting blood loss may also have reduced girls’ iron levels and consequently lead to anemia, particularly in older adolescent girls. For instance, adolescent girls in the United States who have more than three years of menstrual history have been shown to have three times the risk of anemia compared to adolescents with less than three years of menstruation [26].

Nepali adolescent girls, particularly Hindus, experience severe stigmatization around menstruation, which prevents full participation in household chores. This may further limit their access to food and sanitation [27, 28]. Higher rates of anemia have been found among Hindu adolescents in several North Indian studies [29–32]. We found higher odds of anemia in adolescents of Hindu background, although this was not significant. The association between ethnicity and anemia was also not significant suggesting that religion and ethnicity may not be determining factors. Rather, the availability of dietary iron and access to iron-rich foods may be more limited in certain religious or ethnic groups due to food beliefs and eating habits [33]. Further research to assess the effect of food beliefs and nutritional practices on adolescent anemia appears indicated.

In line with previous studies [34–36], eating patterns are major contributors to anemia. We found a higher prevalence of anemia in adolescents who consumed fewer food varieties. The limited consumption of a variety of food sources may reflect household food insecurity from poverty. This may have contributed to the comparatively higher prevalence of anemia in the lowlands (Terai) where, despite fertile and industrial lands, poverty and income disparities are higher than in the other regions [37]. Further, a lack of sanitation facilities in the Terai [11] leads to fecal transmission of helminths, thus inhibiting the internal absorption of nutrients [38, 39]. In this study it was also found that adolescents who did not wear shoes outside were more likely to be anemic. While not wearing shoes can be viewed as a proxy measure of poverty (and thus reflect lack of access to nutritious food), not wearing shoes outside is also a proxy for poor hygiene and increases the chances of hookworm infection [38].

The findings raise a need for context-specific and integrated nutrition programs that address the underlying structural, socio-cultural, environmental and economic determinants of adolescent anemia. An important emphasis should therefore be on integrating nutrition programs with Water Sanitation and Hygiene (WASH) programs in the lowlands, but also with programs that focus on treating hookworm and other helminths. Another strategy could be to introduce a weekly dose regimen of iron supplementation for adolescent girls in schools in order to increase girls’ iron intake [40]. A recent review by Lassi et al [41] showed that iron supplements could significantly improve hemoglobin concentration in adolescents. Efforts should also be steered towards nutritional programs that promote dietary diversity in order to improve the content and bioavailability of micronutrients in household diets [42, 43].

We had a response rate of 99.5%, which is exceptionally high for studies collecting biochemical samples through invasive procedures such as finger prick. The less invasive (and thus less painful) screening method was used to measure capillary hemoglobin, which might have facilitated participation. Lack of routine screening services for anemia in rural areas of Nepal might also have encouraged participation by adolescents.

Despite the strengths that accompany a nationally representative survey with a high response rate, some limitations exist. Our study does not provide regional or district-level estimates of adolescent anemia. We relied on research participants’ perspectives to obtain information on certain determinants, such as dietary habits and sanitary practices. This may have led to some social desirability bias, misclassification and measurement errors. For instance, although a wealth of studies [31, 35, 44–46] have established the effect of economic factors in anemia, no meaningful differences were found between anemia prevalence and the level of household income in the present study. This finding should be interpreted with caution as social desirability bias around family income may have led to some misspecification of the association between the variables. While our study indicated a number of factors significant to iron deficiency anemia, there may be other contributing factors such as thalassemia trait. For instance, the frequency of α-thalassemia trait is high in certain ethnic groups living in malaria-endemic districts of the Terai [47, 48]. This suggests that more detailed hematological testing is warranted on a smaller sample to explore the association with adolescent anemia.

## Conclusion

Overall, anemia is a persisting major public health problem among adolescents in Nepal, particularly in the Terai and among older and female adolescents, where a higher prevalence of anemia was observed. The findings of this study suggest that such high-risk populations should be the focus of context-specific, integrated nutrition intervention programs including nutrition education, mass supplementation with iron and folic acid tablets, treatment of hookworm, and sanitation programs for adolescent girls.

## References

[1] World Health Organization. Nutrition in adolescence–issues and challenges for the health sector: issues in adolescent health and development. Geneva: World Health Organization; 2005.

[2] World Health Organization. Adolescent nutrition: a review of the situation in selected South-East Asian countries. New Delhi: World Health Organization Regional Office for South-East Asia; 2006. Contract No.: SEA-NUT-163.

[3] de Benoist B, McLean E, Egli I, Cogswell M. Worldwide prevalence of anaemia 1993–2005, WHO Global Database on Anaemia Geneva: World Health Organization & Centre for Disease Control and Prevention; 2008.

[4] Prentice AM. Nutrition challenges and issues of relevance to adolescents in low-and middle-income countries. In: Bhutta ZA, Makrides M, Prentice AM, editors. Health and nutrition in adolescents and young women: preparing for the next generation. Nestle Nutrition Institute; 2013 11, Bali. Karger Publisher; 2015(80). p. 49–59.

[5] Sen A, Kanani S. Deleterious functional impact of anemia on young adolescent school girls. Indian pediatr. 2006;43(3):219 16585816

[6] Walker SP, Grantham-McGregor S, Himes JH, Williams S. Adolescent Kingston girls' school achievement: nutrition, health and social factors. Proceedings of the Nutrition Society. 1996;55(1B):333–43. 8832804 10.1079/pns19960033

[7] Bruner AB, Joffe A, Duggan AK, Casella JF, Brandt J. Randomised study of cognitive effects of iron supplementation in non-anaemic iron-deficient adolescent girls. The Lancet. 1996;348(9033):992–6. 10.1016/S0140-6736(96)02341-0 8855856

[8] Li R, Chen X, Yan H, Deurenberg P, Garby L, Hautvast J. Functional consequences of iron supplementation in iron-deficient female cotton mill workers in Beijing, China. Am J Clin Nutr. 1994;59(4):908–13. 10.1093/ajcn/59.4.908 8147338 10.1093/ajcn/59.4.908

[9] Soekarjo D, de Pee S, Bloem M, Tijong R. Socio-economic status and puberty are the main factors determining anaemia in adolescent girls and boys in East Java, Indonesia. Eur J Clin Nutr. 2001;55(11):932 10.1038/sj.ejcn.1601248 11641741 10.1038/sj.ejcn.1601247

[10] Akseer N, Al‐Gashm S, Mehta S, Mokdad A, Bhutta ZA. Global and regional trends in the nutritional status of young people: a critical and neglected age group. Ann N Y Acad Sci. 2017;1393(1):3–20. 10.1111/nyas.13336 28436100 10.1111/nyas.13336

[11] Ministry of Health and Population, New ERA, ICF International Inc. Nepal demographic and health survey 2011. Kathmandu, Nepal: Ministry of Health and Population, New ERA and ICF International, Claverton, Maryland; 2012.

[12] Baral K, Onta S. Prevalence of anemia amongst adolescents in Nepal: a community based study in rural and urban areas of Morang District. Nepal Med Coll J. 2009;11(3):179–82. 20334065

[13] Singh P, Khan S, Ansari M, Mittal R. Anemia amongst adolescent girls and boys attending outpatients and inpatient facilities in far western part of Nepal. Ibnosina Journal of Medicine and Biomedical Sciences. 2013;5(6):330–4.

[14] Sinha A, Karki GMS, Karan KK. Prevalence of anemia amongst adolescents in Biratnagar Morang District Nepal. Int J of Pharm Biol Arch. 2012;3(5).

[15] Central Bureau of Statisticts. Population size, growth and distribution. In: Pathak RS, Lamichhane K, editors. Population monograph of nepal. Kathmandu, Nepal: Government of Nepal, National Planing Comission; 2014.

[16] Sedgwick P. Stratified cluster sampling. BMJ. 2013;347:f7016.

[17] Aryal KK, Mehata RK, Chalise B, Mehata S, Sapkota F, Dhimal M, et al Adolescent nutrition survey in Nepal, 2014. Kathmandu Nepal: Nepal Health Research Council; 2016.

[18] World health Organization. Haemoglobin concentrations for the diagnosis of anaemia and assessment of severity. Geneva: World Health Organization; 2011 (WHO/NMH/NHD/MNM/11.1), Available from: http://www.who.int/vmnis/indicators/haemoglobin.pdf.

[19] Bennett L, Dahal DR, Govindasamy P. Caste ethnic and regional identity in Nepal: further analysis of the 2006 Nepal demographic and health survey. Claverton, Maryland: Macro International Inc; 2008.

[20] Subedi M. Caste system: theories and practices in Nepal. Himalayan Journal of Sociology and Anthropology. 2011;4:134–59.

[21] Bongaarts J, Zimmer Z. Living arrangements of older adults in the developing world: an analysis of demographic and health survey household surveys. J Gerontol B Psychol Sci Soc Sci. 2002;57(3):S145–S57. 11983741 10.1093/geronb/57.3.s145

[22] World Health organization. Growth reference 5–19 years [Internet]. Geneva: World health Organization; 2007 [cited 2018 Aug 8]. Available from: http://www.who.int/growthref/who2007_bmi_for_age/en/.

[23] Ruel MT. Operationalizing dietary diversity: a review of measurement issues and research priorities. The Journal of Nutrition. 2003;133(11):3911S–26S. 14672290 10.1093/jn/133.11.3911S

[24] Midi H, Sarkar S, Rana S. Collinearity diagnostics of binary logistic regression model. Journal of Interdisciplinary Mathematics. 2010;13(3):253–67.

[25] DeRose LF, Das M, Millman SR. Does female disadvantage mean lower access to food? Population and Development Review. 2000;26(3):517–47.

[26] Sekhar DL, Murray-Kolb LE, Kunselman AR, Weisman CS, Paul IM. Association between menarche and iron deficiency in non-anemic young women. PloS One. 2017;12(5):e0177183 10.1371/journal.pone.0177183 28486542 10.1371/journal.pone.0177183 PMC5423639

[27] Crawford M, Menger LM, Kaufman MR. ‘This is a natural process’: managing menstrual stigma in Nepal. Culture, Health & Sexuality. 2014;16(4):426–39. 10.1080/13691058.2014.887147 24697583

[28] Ranabhat C, Kim C-B, Choi EH, Aryal A, Park MB, Doh YA. Chhaupadi culture and reproductive health of women in Nepal. Asia Pac J Public Health. 2015;27(7):785–95. 10.1177/1010539515602743 26316503 10.1177/1010539515602743

[29] Verma P, Singh S, Ghildiyal A, Kumar A, Krishna A. Prevalence of anaemia in adults with respect to socio-demographic status, blood groups and religion in north indian population. Int J Bio Med Res. 2012;3(4):2422–8.

[30] Chib R. Statistics on Prevalence of anaemia in adults with respect to age, gender and religion: a study of jammu city. Indian J Appl Res 2014;4(9):635–7.

[31] Lokare PO, Karanjekar VD, Gattani PL, Kulkarni AP. A study of prevalence of anemia and sociodemographic factors associated with anemia among pregnant women in Aurangabad city, India. Annals of Nigerian Medicine. 2012;6(1):30–4.

[32] Bharati P, Chakrabarty S, Pal M. Prevalence of Anemia and Its Determinants Among Nonpregnant and Pregnant Women in India. Asia Pac J Public Health 2008;20(4):347–59. 10.1177/1010539508322762 19124329 10.1177/1010539508322762

[33] Madjdian DS, Azupogo F, Osendarp SJ, Bras H, Brouwer ID. Socio-cultural and economic determinants and consequences of adolescent undernutrition and micronutrient deficienceis in LMICs: a systematic narrative review. Ann N Y Acad Sci. 2018;1416(1):117–39.

[34] Alaofè H, Burney J, Naylor R, Taren D. Prevalence of anaemia, deficiencies of iron and vitamin A and their determinants in rural women and young children: a cross-sectional study in Kalalé district of northern Benin. Public Health Nutrition. 2017:1–11. 10.1017/S1368980016003608 PMC10261276 28120735

[35] Gebre A, Mulugeta A. Prevalence of anemia and associated factors among pregnant women in North Western zone of Tigray, Northern Ethiopia: a cross-sectional study. J Nutr Metab. 2015;2015. 10.1155/2015/165430 PMC4475559 26137321

[36] Obse N, Mossie A, Gobena T. Magnitude of anemia and associated risk factors among pregnant women attending antenatal care in Shalla Woreda, West Arsi Zone, Oromia Region, Ethiopia. Ethiop J Health Sci. 2013;23(2):165–73. 23950633 PMC3742894

[37] Pokharel T. Poverty in Nepal: Characteristics and Challenges. Journal of Poverty, Investment and Development. 2015;11.

[38] Parajuli R, Fujiwara T, Umezaki M, Konishi S, Takane E, Maharjan M, et al Prevalence and risk factors of soil-transmitted helminth infection in Nepal. Trans R Soc Trop Med Hyg. 2014;108(4):228–36. 10.1093/trstmh/tru013 24488979 10.1093/trstmh/tru013

[39] Janmohamed A, Karakochuk CD, McLean J, Green TJ. Improved Sanitation Facilities are Associated with Higher Body Mass Index and Higher Hemoglobin Concentration Among Rural Cambodian Women in the First Trimester of Pregnancy. Am J Trop Med Hyg. 2016;95(5):1211–5. 10.4269/ajtmh.16-0278 27549631 10.4269/ajtmh.16-0278 PMC5094240

[40] Darnton-Hill I. Solving the micronutrient problem in the Asia Pacific region. Asia Pac J Clin Nutr. 1998;7:245–55. 24393679

[41] Lassi ZS, Moin A, Das JK, Salam RA, Bhutta ZA. Systematic review on evidence‐based adolescent nutrition interventions. Ann N Y Acad Sci. 2017;1393(1):34–50. 10.1111/nyas.13335 28436101 10.1111/nyas.13335

[42] Rao S, Joshi S, Bhide P, Puranik B, Asawari K. Dietary diversification for prevention of anaemia among women of childbearing age from rural India. Public Health Nutrition. 2014;17(04):939–47. 23594695 10.1017/S1368980013001006 PMC10282298

[43] Gibson RS, Hotz C. Dietary diversification/modification strategies to enhance micronutrient content and bioavailability of diets in developing countries. Br J Nutr. 2001;85(S2):S159–S66. 11509105 10.1079/bjn2001309

[44] Melku M, Addis Z, Alem M, Enawgaw B. Prevalence and predictors of maternal anemia during pregnancy in Gondar, Northwest Ethiopia: an institutional based cross-sectional study. Anemia. 2014;2014. 10.1155/2014/108593 PMC3942101 24669317

[45] Balarajan Y, Ramakrishnan U, Özaltin E, Shankar AH, Subramanian S. Anaemia in low-income and middle-income countries. The Lancet. 2012;378(9809):2123–35. 10.1016/S0140-6736(10)62304-5 21813172

[46] Bharati P, Som S, Chakrabarty S, Bharati S, Pal M. Prevalence of anemia and its determinants among nonpregnant and pregnant women in India. Asia Pac J Public Health. 2008;20(4):347–59. 10.1177/1010539508322762 19124329 10.1177/1010539508322762

[47] Modiano G, Morpurgo G, Terrenato L, Novelletto A, Di Rienzo A, Colombo B, et al Protection against malaria morbidity: near-fixation of the α-thalassemia gene in a Nepalese population. Am J Hum Genet. 1991;48(2):390 1990845 PMC1683029

[48] Sakai Y, Kobayashi S, Shibata H, Furuumi H, Endo T, Fucharoen S, et al Molecular analysis of α-thalassemia in Nepal: correlation with malaria endemicity. J Hum Genet. 2000;45(3):127–32. 10.1007/s100380050198 10807536 10.1007/s100380050198

